# Isolated perforation of a duodenal diverticulum following blunt abdominal trauma

**DOI:** 10.4103/0974-2700.58656

**Published:** 2010

**Authors:** Matthew J Metcalfe, Tanwir G Rashid, Richard le R Bird

**Affiliations:** Department of Surgery, Barnet Hospital, Barnet and Chase Farm NHS Trust, Wellhouse Lane, Barnet Herts EN5 3DJ, UK

**Keywords:** Duodenal diverticulum, blunt trauma, perforation

## Abstract

Only 10% of duodenal diverticula are symptomatic. We present the case of a man who fell from a height of 6 ft, landing on his abdomen and presenting 4 h later with severe back pain and a rigid abdomen. At laparotomy, a perforated retroperitoneal duodenal diverticulum was found and repaired with an omental patch. No other injury was noted. Not only is this perforation unusual, but the absence of other injuries sustained during this minor blunt trauma makes this case unique. This case highlights the need for a high index of suspicion when managing patients with back or abdominal pain following minor trauma.

## INTRODUCTION

A diverticulum is an outpouching of a hollow viscus and can occur in the bladder, heart, or anywhere throughout the gastrointestinal (GI) tract. Examples include gastric, jejunal, Meckel, and colonic diverticula. Duodenal diverticula were first described in 1710.[1] About 60–95% are seen in the second part of the duodenum, usually within 2.5 cm of the ampulla.[2,3] Only 10% of duodenal diverticula are symptomatic, presenting with steatorrhea, abdominal pain, GI bleeding,[3] perforation, biliary/pancreatic obstruction, intestinal obstruction, and diverticulitis.[2] Intraluminal duodenal diverticula have been described.[4]

Just over 100 cases of perforation of a duodenal diverticulum have been reported in the literature. Most occur spontaneously at the second part of the duodenum and 70% perforate retroperitoneally.[5] We present a case of an isolated duodenal diverticulum perforation following blunt abdominal trauma. The case was managed surgically.

## CASE REPORT

**Figure 1:**
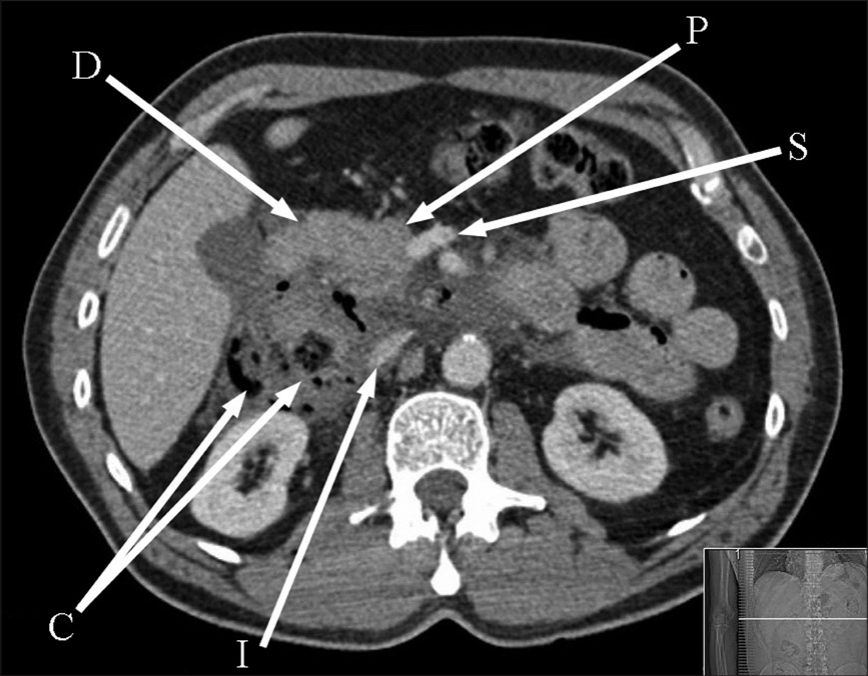
Computed tomography, axial image, demonstrating a retroperitoneal fluid- and air-filled collection posterior to the second part of the duodenum. (P = pancreatic head, S = splenic vein, I = inferior vena cava, C = air- and fluid-filled collection, D = duodenum)

A 58-year-old man fell off a ladder from a height of 6 ft, landing on his abdomen. With the exception of a few abrasions, he appeared unscathed. Four hours later, however, he developed severe back pain and attended the accident and emergency department, still able to walk independently. His past medical history included angina as well as a duodenal diverticulum that had been diagnosed endoscopically 20 years previously. On arrival he was pale, sweating, and apyrexial. He had blood pressure of 130/80 mmHg and a pulse rate of 104/min. Abdominal examination revealed generalized peritonitis. Blood tests revealed total white cell count of 19 × 10^9^/l and hemoglobin of 12 g/dl. A computed tomography (CT) scan showed an 8 × 7 × 7 cm fluid- and gas-filled collection arising from the junction of the 2^nd^ the 3^rd^ parts of the duodenum. The findings were consistent with retroperitoneal duodenal perforation [Figure 1].

**Table 1:** Duodenal injury severity according to the American Association for the Surgery of Trauma[6]

Grade	Injury	Description
I	Hematoma	Involving single portion of duodenum
	Laceration	Partial thickness, no perforation
II	Hematoma	Involving more than one portion
	Laceration	Disruption <50% of circumference
III	Laceration	Disruption of 50–70% of circumference of D2
		Disruption of 50–100% of circumference of D1, D3, and D4
IV	Laceration	Disruption of >75% of circumference of D2
		Involving ampulla or common bile duct
V	Laceration	Massive disruption of pancreatico-duodenal complex
	Vascular	Devascularization of the duodenum

ADVANCE ONE GRADE FOR MULTIPLE INJURIES TO THE DUODENUM

Laparotomy was done and revealed a perforated duodenal diverticulum at the posterior aspect of the junction of the 2^nd^ the 3^rd^ parts of the duodenum, with a 200 ml retroperitoneal hematoma mixed with bilious fluid behind the pancreatic head. Kocher maneuver enabled duodenal mobilization and direct access to the diverticulum. Repair involved excision of the diverticulum and primary closure of the duodenum with interrupted 2-0 polydioxanone submucosal sutures. An omental patch was sutured in place over the closure. There was no evidence of injury to any other abdominal organ.

He made a good postoperative recovery, but on day 8 he became confused and developed a wound infection and septicemia; this led to a wound dehiscence and, subsequently, incisional hernia. On day 11 following surgery his hemoglobin fell to 8.5 g/dl due to a gastrointestinal bleed, likely from the diverticulum edge. Endoscopy was withheld and the bleeding stopped spontaneously.

## DISCUSSION

The frequent coexistence of duodenal and pancreatic injuries has led to a combined classification of these injuries. However, Moore *et al*. have classified isolated traumatic duodenal injuries[6] and, as per their classification, our patient had a grade II injury.

Spontaneous duodenal diverticulum perforation has been reported earlier.[7] Traumatic — as opposed to iatrogenic (e.g., endoscopic) — perforation is unusual. Adding to the uniqueness of this case is the absence of injury to any of the other organs. This relatively minor degree of trauma resulted in an isolated but significant and life-threatening injury, with pooling of activated digestive enzymes, likely including amylase, in the retroperitoneal space. Clinicians must have a low threshold for investigating patients following such minor trauma, and non-musculoskeletal causes must also be considered when managing traumatic back pain.

Blunt injury to the abdomen commonly affects the liver, spleen, and kidneys. Small bowel injury is also known to occur as a result of deceleration forces causing tears near fixed points of attachment. In our patient, we suspect that compression of the diverticulum at the point where it passes over the 2^nd^ and 3^rd^ lumbar vertebra occurred during deceleration, resulting in its rupture. The peritoneal attachments give anchorage to the first part of the duodenum and the duodenojejunal junction; in between these two points, the retroperitoneal duodenum is sandwiched between the pancreas, the right psoas muscle, the inferior vena cava, and the right kidney posteriorly, and only the transverse colon and the superior mesenteric vessels anteriorly. Thus, on impact, this duodenal diverticulum was compressed between the anterior and posterior structures, which likely resulted in its perforation. However, no injury occurred at the duodenal anchorage points. No injury was sustained to the right kidney or to its hilar vessels lying in close proximity.

Our surgical management of this patient is similar to that reported by other authors,[8] though conservative management (for grade 1 injuries), gastrectomy with a Bilroth II gastrojejunostomy (for grade III injuries), and pancreaticoduodenectomy (for grades IV and V injuries) are also documented.[9] It is possible that percutaneous drainage could have controlled the local sepsis and helped drain the active enzymes in the retroperitoneal space, but any attempts at conservative management would also run the risk of complications such as pancreatic and duodenal fistula formation and recurrence of the collections. Thus, conservative measures should probably be reserved for those not fit for major surgery or those with other significant injuries. Diagnostic laparoscopy does not appear to be better that other imaging modalities for detecting duodenal injury[10] although in expert hands, following exploration of the retroperitoneal space, it may be possible to carry out laparoscopic Kocherization and subsequent duodenal repair with an omental patch. Alternatively, a direct anastomosis of a Roux-en-Y loop sutured over the duodenal defect in an end-to-side fashion could have been undertaken, particularly if the defect were large.[9] Perioperative mortality for symptomatic duodenal diverticula ranges from 3% to 31%,[6,11] and the morbidity is around 15%. In this patient, wound infection, incisional hernia, and gastrointestinal bleeding were the three complications that we came across following surgical repair. A duodenal injury and a time to surgery of greater than 18 h is an adverse prognostic factor,[12] as are age, hypotension on arrival, a negative base deficit, a lower initial arterial pH, and an associated inferior vena cava injury.[13]

Surgery for perforated duodenal diverticula is thus recommended in emergency presentations or for intractable symptoms only.[11]
